# Obituary

**Published:** 1894-04

**Authors:** 


### Obituary.

Dr. Hampton Eugene Hill died at his home in Saco, Maine, January 9, 1894, in the forty-fifth year of his age. He had been in ill-health for some time, partly because of the severe strain of an exacting professional work, but principally because of the death of his wife some two years ago, from the shock of which he never recovered.

Dr. Hill was one of the most eminent abdominal surgeons in his region of country, and had attained a fame even in his younger years that might well be the envy of any man in advanced life. He performed twelve ovariotomies before he ever saw the operation done by another, and had not even seen the abdomen opened for any cause, except on the cadaver. He undertook and carried through to success some of the most desperate cases, under environments of great discouragement and such as would appall a less courageous operator. He was often called great distances into the country and after operating would remain with the patient until she was out of danger, himself attending to every detail of after-treatment. In the region where he practised it was often difficult to obtain competent nurses, and rather than run the risk of failure by faulty after-management he adopted this course. In one case he removed a solid tumor, weighing forty-seven pounds, and put the patient to bed in a state of collapse. After a time, however, under proper stimulation she revived and ultimately made a good recovery. In his account of this case Dr. Hill says: "I spent three weeks with this patient away from home and did nothing else but take care of her with the nurse. During the first nine days I did not leave the house but attended to every detail of care and diet myself, by day and night." Dr. Hill was a most loveable character, amiable as a woman, but when occasion required he was as courageous as a lion. He was modest almost to diffidence, a man of simple tastes and few words, but a strong character, made doubly so by the environment of his field of labor. He will be greatly missed by a devoted clientèle and a large group of personal relatives and friends.

---

Dr. Alexander Dunlap, of Springfield, Ohio, died at his home in that city on Friday, February 6, 1894, aged 79 years. Dr. Dunlap was for many years one of the most distinguished surgeons west of the Alleghany mountains, and his death removes one of the most conspicuous medical men of his time. In 1843, not knowing that Clay, of England, and Atlee, of Philadelphia, had antedated him for a few months, he removed an ovarian tumor, basing his diagnosis entirely on the traditions of McDowell's cases, thus reviving a surgical art that for more than thirty years had been lost, but that has now become one of the most useful and successful branches of major surgery extant. Surrounded by a few neighboring physicians, Dunlap, on the 17th of September, 1843, successfully removed an ovarian tumor weighing forty-five pounds, the first ovariotomy made west of the Alleghany mountains subsequent to McDowell's time. A few weeks later the patient died of complications not due to the operation, but Dunlap was denounced by his contemporaries for having undertaken a brutal and useless operation. He, however, outlived this denunciation and subsequently made over 400 abdominal sections. He also operated for stone, having successfully removed a calculus weighing twenty ounces. He also three times removed the under jaw, ligated the common carotid artery, and removed the clavicle.

Dr. Dunlap has taken a conspicuous part in the medical societies, local, state and national, and has left a fame that will ever remain associated with the history of medicine of the nineteenth century.

